# Effect of Light Sources on Transmittance of Commercially Available Contact Lenses

**DOI:** 10.7759/cureus.62093

**Published:** 2024-06-10

**Authors:** Ravindra K Gupta, Mohammed A Alzayed, Abdulrahman A Aba Alkhayl, Thafer S Bedaiwi

**Affiliations:** 1 Optometry Department, College of Applied Medical Sciences, King Saud University, Riyadh, SAU; 2 Diagnostic Imaging Department, King Khaled Eye Specialist Hospital, Riyadh, SAU

**Keywords:** visible light, ultraviolet ray, transmission, spectrophotometry, contact lenses

## Abstract

Background: Previous studies have suggested that light rays may interact with contact lenses, potentially affecting their transmittance.

Aim: This study aimed to investigate the effects of visible and ultraviolet (UV)-A light sources on the transmittance of some commercially available daily, weekly, and monthly contact lenses.

Methods: Nine commercially available soft contact lenses were irradiated with a solar simulator, light-emitting diode (LED) source, laser source, and UV-A source. The average transmittance of the tested lenses before and after irradiation in the UV, visible, and infrared light wavelength ranges was determined using an Agilent UV-visible spectrophotometer, model 8453.

Results: The results showed a partial or complete block of UV transmission at the UV-B region (300 nm) and the UV-A region (355 nm) by the Bio true daily contact lens, as well as the Acuvue Oasys, Avaira, and Biomedics 55 weekly lenses. At the visible region (555 nm), irradiation of the contact lenses by different light sources resulted in reduced light transmittance. At the infrared region (900 nm), the weekly and monthly contact lenses partially blocked infrared transmission, while the daily lenses showed either increased or decreased infrared transmission.

Conclusions: Solar and artificial lighting, as well as high-powered lasers, constitute a major concern on the contact lenses' light transmission and optical properties. It is essential to develop soft contact lenses that have photoprotective properties while maintaining visible light transmittance.

## Introduction

Contact lenses have gained popularity over the years. They are curved plastic disks placed directly on the surface of the cornea through the tear film. To ensure a proper fit, the posterior surface of the contact lens should have enough aspheric curvature to closely conform to the contour of the anterior surface of the cornea [1].

Contact lenses come in different shapes and sizes, such as hard, soft, semisoft, and gas-permeable types. Soft contact lenses are mainly made of 2-hydroxyethyl methacrylate polymers, with or without silicon, to achieve a specific water content. There is a wide variety of soft contact lenses available commercially, and they can be used daily, weekly, or monthly [2].

Contact lenses are primarily used to correct refractive errors such as myopia, hypermetropia, astigmatism, anisometropia, aphakia, and presbyopia. They can also be used for cosmetic purposes, such as changing the color of the eye or camouflaging a blind eye with a corneal scar [3]. Additionally, contact lenses have therapeutic benefits for optimal drug delivery in various ophthalmic diseases [4]. Ophthalmologists may utilize a variety of contact lenses, including gonioscopy lenses, in conjunction with slit lamps for diagnostic purposes [5].

There are three known types of light spectrums: visible light, ultraviolet (UV), and infrared rays. Visible light has a wavelength range between 400 nm and 700 nm. UV rays cover a wavelength range of 100-400 nm and are divided into three regions: UV-A (315-400 nm), UV-B (280-315 nm), and UV-C (100-280 nm). Infrared rays have a wavelength range of 700 nm to 1 mm [6].

There are several sources of light. Visible light can be produced by a solar simulator, while UV-A rays are produced by a black light lamp. Electroluminescent sources such as light-emitting diodes (LEDs) and thermal sources such as tungsten or tungsten-halogen lamps emit light at sufficiently high temperatures [7]. Semiconductor Nd-YAG lasers are sources of monochromatic and bright light [8].

Transmittance is an optical property of contact lenses that represents the amount of refracted light. It has been suggested that light rays might interact with the contact lenses while passing through them depending on the lens material, intensity of the incident light, and environmental conditions [9].

The present study aimed to examine the impact of visible and UV-A light sources on the transmittance of some commercially available daily, weekly, and monthly contact lenses.

## Materials and methods

Study design, setting, and date

This comparative observational study was conducted at the College of Applied Medical Sciences, King Saud University, Riyadh, Saudi Arabia from May 2017 to April 2018.

Contact lenses

Nine commercially available daily (N=3), weekly (N=3), and monthly (N=3) soft contact lenses with a power range between -1D and -10D were chosen. Bare contact lenses without irradiation were used as a control. The lenses belong to different companies, and the necessary information is illustrated in Table 1.

**Table 1:** Commercially Available Daily, Weekly, and Monthly Soft Contact Lenses Used in the Present Study UV, ultraviolet.

Company	Type	Power (D)	Base curve (mm)	Diameter (mm)	Lens materials	UV protection
Bio true, BAUSCH+LOMB	Daily	-6	8.6	14.2	Nesofilicon A, WC 78%, OP 42 Dk/t	Unknown
Dailies Aqua Comfort Plus, Alcon	Daily	-10	8.7	14	Nefilcon A, WC 69%, OP 26Dk/t	Unknown
Dailies Total, Alcon	Daily	-6	8.5	14.1	67% Delefilcon A, WC 80%, OP 156 Dk/t	Unknown
Avaira, CooperVision	Weekly	-1.75	8.5	14.2	Etafilcon A, WC 46%, OP 125 Dk/t	75%UVA, 99%UVB
Acuvue, Oasys, Johnson & Johnson	Weekly	-1	8.4	14	Senofilcon A, WC 38%, OP 147Dk/t	96%UVA, 99.9%UVB
Biomedics 55 Evolution, CooperVision	Weekly	-1.50	8.6	14.20	Ocufilcon D, WC 55%, OP 27 Dk/t	Unknown
PureVision2, BAUSCH+LOMB	Monthly	-1.50	8.6	14	Balafilcon A, WC 36%, OP 130 Dk/t	Unknown
Biofinity CooperVision	Monthly	-2	8.6	14	Comfilcon A, WC 48%, OP 160 Dk/t	No
Frequency55, CooperVision	Monthly	-1	8.7	14.4	Methafilcon A, WC 55%, OP 20 Dk/t	Unknown

Light sources

Light illumination was carried out using commercially available light sources. A class BBA small-area solar simulator (PV measurements, Inc., Point Roberts, WA, USA) was used to produce simulated sunlight of 1 Sun (100 mW/cm^2^). Additionally, an IKEA LED bulb (model KMV-040-030-BS, 2 W) with a luminous flux of 88 lm, a black light tube of 4 W for UV-A irradiation, and a semiconductor laser (model Photon) with a wavelength of 650 nm and 1 mW were also used.

Procedure

**Figure 1:**
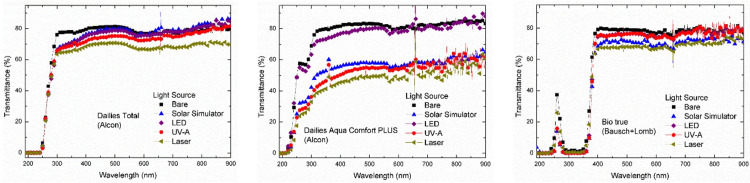
Transmittance Spectra of Bare and Irradiated Daily Contact Lenses UV, ultraviolet; LED, light-emitting diode.

**Figure 2:**
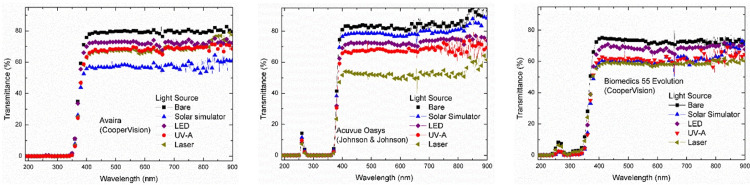
Transmittance Spectra of Bare and Irradiated Weekly Contact Lenses UV, ultraviolet; LED, light-emitting diode.

**Figure 3:**
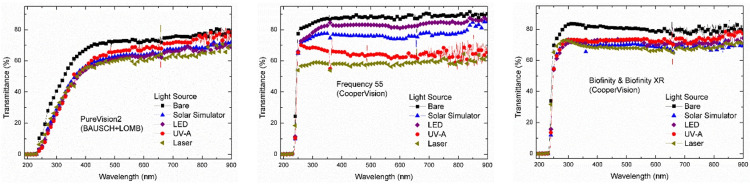
Transmittance Spectra of Bare and Irradiated Monthly Contact Lenses UV, ultraviolet; LED, light-emitting diode.

Visible rays were irradiated on contact lenses by a solar simulator, an LED source, and a laser source, while UV-A rays were irradiated by a UV-A source. The distance between the light source and the contact lens was 10 cm. Then, using an Agilent UV-visible spectrophotometer (model 8453), the transmittance spectra of the nine light-irradiated and bare daily (Figure 1), weekly (Figure 2), and monthly (Figure 3) contact lenses were determined.

## Results

In the present study, monitoring the transmittance of the bare and the irradiated daily, weekly, and monthly soft contact lenses at different wavelengths revealed the following.

At the UV-B region (300 nm), the Bio true lenses completely blocked the UV-B rays. The bare Dailies Aqua Comfort Plus lenses showed an average transmittance of 69%, which was reduced to 63% after visible rays irradiation from the LED bulb, 42% after sunlight irradiation, 36% after UV-A irradiation, and 33% after laser irradiation. The bare samples of Dailies Total lenses showed an average transmittance of 76%, while visible and UV-A irradiation on these lenses reduced transmittance to about 65%, and laser irradiation reduced transmittance to about 63%. For the bare and irradiated weekly lenses, transmittance values were nearly zero, indicating complete blocking of the UV-B rays. The bare samples of the monthly contact lenses showed transmittance of 83% for Biofinity, 40% for PureVision2, and 85% for Frequency 55. The irradiated Biofinity lenses observed showed a reduction of nearly 72% in transmittance. Irradiated PureVision2 lenses exhibited a reduction in transmittance between 25% and 30%. The transmittance of the irradiated Frequency 55 lenses was approximately 78% for the LED source, 75% for the solar simulator, 79% for the UV-A source, and 59% for the laser source (Table 2).

**Table 2:** Comparison of the Average Transmittance (%) at Wavelengths of 300 nm (UV-B Region) Data between parentheses corresponds to the standard error value.

Contact lens		Bare	Light source			
			Solar simulator	LED	UV-A	Laser
Daily	Bio true	1.24 (0.005)	0.03 (0.01)	0.08 (0)	0.04 (0)	0.48 (0)
	Dailies Aqua Comfort Plus	69 (0)	42.2 (0.1)	63.25 (0.15)	36.3 (0.1)	32.89 (0.095)
	Dailies Total	76.55 (0.45)	66.35 (0.05)	64.6 (0.1)	65.86 (0.005)	63.5 (0)
Weekly	Acuvue Oasys	0.01 (0)	0.01 (0)	0.01 (0)	0.01 (0)	0.01 (0)
	Avaira	0.05 (0.045)	0.02 (0)	0.01 (0.005)	0.01 (0)	0.01 (0)
	Biomedics 55 Evolution	1.13 (0.015)	0.12 (0)	0.15 (0.005)	0.16 (0.005)	0.94 (0.003)
Monthly	Biofinity	83.1 (0)	72.7 (0.5)	71.40 (0.046)	73 (0.3)	72.43 (0.195)
	PureVision2	40.6 (0)	28.99 (0.025)	26.79 (0.055)	25.51 (0.09)	30.36 (0.095)
	Frequency55	84.75 (0.55)	75.55 (0.05)	77.9 (0.2)	68.7 (0)	58.78 (0.105)

At the UV-A region (355 nm), the bare Bio true (daily), Acuvue Oasys (weekly), Avaira (weekly), and Biomedics 55 (weekly) lenses exhibited average transmittance between 0% and 19%, indicating complete or partial blocking of the UV-A rays. Alternatively, the bare samples of Dailies Aqua Comfort Plus and Dailies Total lenses showed transmittance of 80% and 78%, respectively. The irradiation of light rays reduced the transmittance of Dailies Aqua Comfort Plus lenses in the following order, about 73% for LED source, 53% for solar simulator, 46% for UV-A source, and 41% for laser source. The irradiated Dailies Total lenses had the following order in transmittance, about 71% for LED source and solar simulator, 69% for UV-A source, and 66% for laser source. The bare samples of Biofinity monthly lenses had a transmittance of approximately 82%, which was reduced to 72% after irradiation from LED and UV-A sources, 70% for solar simulators, and 69% for laser sources. The PureVision2 monthly lenses had transmittance of about 60% and reduced to 48% after visible light irradiation by a solar simulator, and 46% after irradiation by LED, UV-A, and laser light sources. Another brand of monthly lenses, Frequency 55 had a transmittance of about 88%; however, the irradiation reduced its transmittance to ~82% for the LED source, ~77% for the solar simulator, 66% for UV-A source, and ~59% for laser source (Table 3).

**Table 3:** Comparison of the Average Transmittance (%) at Wavelengths of 355 nm (UV-A Region) Data between parentheses corresponds to the standard error value.

Contact lens		Bare	Light source			
			Solar simulator	LED	UV-A	Laser
Daily	Bio true	4.52 (0.02)	0.5 (0)	0.73 (0.005)	0.5 (0)	2.29 (0.02)
	Dailies Aqua Comfort Plus	80.05 (0.05)	52.9 (0.1)	73.4 (0.1)	46.55 (0.05)	41.2 (0.1)
	Dailies Total	77.91 (0.065)	71.25 (0.05)	71.09 (0.205)	69.2 (0)	65.6 (0)
Weekly	Acuvue Oasys	0.10 (0.005)	0.05 (0.04)	0.11 (0)	0.08 (0.005)	0.04 (0.005)
	Avaira	4.59 (0.005)	3.8 (0)	4.97 (0.045)	2.08 (0)	2.53 (0.13)
	Biomedics 55 Evolution	19 (0.1)	8.06 (0.01)	9.66 (0.025)	8.77 (0.01)	14.89 (0.001)
Monthly	Biofinity	82.6 (0)	70.34 (0.34)	71.66 (0.025)	72.4 (0.3)	69.17 (0.13)
	PureVision2	59.6 (0)	48.11 (0.015)	45.45 (0.045)	45.89 (0.11)	46.32 (0.12)
	Frequency55	87.7 (0.2)	77.55 (0.05)	82.5 (0.2)	66 (0)	58.78 (0.105)

At the visible region (555 nm), the bare samples of daily contact lenses exhibited average transmittance between 78% and 82%. The light irradiation reduced the transmittance of Bio True lenses to 76% for LED and UV-A sources, 71% for solar simulators, and 68% for laser sources. The irradiated Dailies Total lenses followed the following order of transmittance: 78% for the solar simulator, 77% for the LED source, 74% for the UV-A source, and 68% for the laser source. The irradiation on Dailies Aqua Comfort Plus lenses reduced the transmittance to 79% for the LED source, 57% for the solar simulator, 55% for the UV-A source, and 49% for the laser source. The weekly lenses, Acuvue Oasys, Avaira, and Biomedics 55 Evolution had average transmittance between 72% and 81%. The irradiated Acuvue Oasys lenses showed transmittance between 50% (laser source) and 78% (solar simulator). The irradiated Avaira lenses showed the following order in transmittance: 73% for LED source, 69% for UV-A source, 67% for laser source, and 58% for solar simulator. The Biomedics 55 lenses showed an average transmittance of 72% for bare samples, 68% for LED source, 60% for solar simulator and UV-A source, and 58% for laser source. In the monthly contact lenses, Biofinity showed an average transmittance of 80% for bare samples, 73% for LED and UV-A sources, 70% for solar simulators, and ~67% for laser sources. In the PureVision2 lenses, transmittance was 73% for bare samples, 67% for UV-A source, 63% for LED source, 64% for solar simulator, and 61% for laser source. The Frequency 55 lenses showed a transmittance value of 88% for bare samples, 82% for LED source, 75% for solar simulator, 64% for UV-A source, and 59% for laser source (Table 4).

**Table 4:** Comparison of the Average Transmittance (%) at Wavelengths of 555 nm (Visible Region) Data between parentheses corresponds to the standard error value.

Contact lens		Bare	Light source			
			Solar simulator	LED	UV-A	Laser
Daily	Bio true	78.64 (0.03)	71.0 (0.0)	76.18 (0.025)	76.1 (0.13)	68.24 (0.02)
	Dailies Aqua Comfort Plus	81.85 (0.05)	57.5 (0.1)	79.4 (0.1)	54.84 (0.01)	49.2 (0.1)
	Dailies Total	78.85 (0.15)	78.17 (0.055)	76.98 (0.01)	73.89 (0)	68.35 (0.05)
Weekly	Acuvue Oasys	81.49 (0.03)	77.8 (0)	72.2 (0)	67.38 (0.335)	50.69 (0.52)
	Avaira	79.72 (0.13)	57.66 (0)	73.3 (0.31)	69.12 (0.5)	67.7 (0)
	Biomedics 55 Evolution	72.65 (0.55)	59.95 (0.15)	67.85 (0.05)	60.74 (0.105)	58.36 (0.193)
Monthly	Biofinity	80 (0)	69.75 (0.35)	72.44 (0.006)	73.45 (0.15)	67.48 (0.155)
	PureVision2	73 (0)	64.01 (0.03)	62.93 (0.07)	67.36 (0.04)	61.16 (0.22)
	Frequency55	87.85 (0.05)	75.25 (0.002)	82.4 (0.2)	63.7 (0)	58.62 (0.11)

At the infrared region (900 nm), the bare samples of Bio true daily lenses exhibited an average transmittance of 77%, which was increased to 79% for LED and UV-A sources, but it was reduced to 71% for solar simulators, and 73% for laser source. For the Dailies Aqua Comfort Plus, the average transmittance of bare samples was approximately 83%, which increased to 87% for LED sources and reduced to 65% for solar simulators, and 61% for UV-A and laser sources. For the Dailies Total, the average transmittance of bare samples was about 80% and it was increased to 86% after solar simulator irradiation, 85% for the LED source and 81% for the UV-A source, and reduced to 70% for the laser source. The Acuvue Oasys weekly lens showed transmittance in the following order: 96% for bare sample, 89% for solar simulator, 74% for LED source, 69% for UV-A source, and ~61% for laser source. Avaira bare samples displayed ~ 80% transmittance that was reduced in the following order: 77% for laser source, 72% for LED source, 69% for UV-A source, and 61% for solar simulator. Biomedics 55 Evolution lenses showed the transmittance as 71% for bare samples, 71% for the solar simulator, 68% for LED source, 64% for UV-A source, and 60% for laser source. For the bare samples of the monthly contact lenses, transmittance was 80% for Biofinity, 78% for PureVision2, and 90% for Frequency 55. The irradiated samples of Biofinity showed transmittance of 77% for UV-A source, 74% for LED source, 71% for laser source, and 69% for solar simulator. The transmittance of irradiated PureVision2 had a value of 76% for the UV-A source, 72% for the solar simulator, 70% for the laser source, and ~69% for the LED source. Frequency 55 lenses showed transmittance of 86% for LED source, 85% for solar simulator, 65% for UV-A source, and 62% for laser source (Table 5).

**Table 5:** Comparison of the Average Transmittance (%) at Wavelengths of 900 nm (Infrared Region) Data between parentheses corresponds to the standard error value.

Contact lens		Bare	Light source			
			Solar simulator	LED	UV-A	Laser
Daily	Bio true	77.47 (0.305)	71.5 (0.5)	79.2 (0.01)	78.9 (0.06)	73.44 (0.025)
	Dailies Aqua Comfort Plus	83.09 (0.145)	64.7 (0.2)	87.7 (0.28)	60.75 (0.65)	61.2 (0.5)
	Dailies Total	79.94 (0.015)	86.55 (0.05)	84.65 (0.25)	81.45 (0.15)	70 (0.2)
Weekly	Acuvue Oasys	95.82 (0.125)	88.69 (0.28)	73.9 (0)	69.28 (0.125)	61.28 (0.245)
	Avaira	79.81 (0.38)	61.06 (0)	72.56 (0.255)	68.94 (0.39)	77.37 (0)
	Biomedics 55 Evolution	71.5 (0.5)	71.3 (0.3)	67.8 (0)	64.53 (0.155)	59.65 (0.535)
Monthly	Biofinity	79.6 (0)	69.25 (0.05)	73.94 (0.375)	76.8 (0.3)	70.93 (0.16)
	PureVision2	78.1 (0)	72.31 (0.18)	68.93 (0.135)	75.74 (0.455)	70.53 (0.45)
	Frequency55	89.95 (0.25)	85.25 (0.125)	86.25 (0.25)	65.5 (0)	62.48 (0.085)

## Discussion

To ensure good eye health and quality vision, contact lenses should have low UV transmittance and high visible light transmittance. Contact lenses are multifunctional biomaterials that interact with various system environments [10]. In recent years, exposure to artificial lights, such as LED lamps, has significantly increased, which may have deleterious effects on most eye tissues [11].

This study measured the transmittance of nine soft contact lenses in the UV, visible, and infrared wavelength ranges before and after visible and UV-A light irradiation. The tested light sources reduced the contact lens transmission of both the UV and visible lights, modifying their optical properties.

UV light has more energy than visible light and can cause damage to ocular tissues. Long-term exposure to UV rays can lead to cataracts, pterygium, or photokeratitis [12]. To protect human eyes from the harmful effects of ultraviolet radiation, manufacturers have incorporated UV-blocking monomers into contact lenses [13].

In this study, it was found that only Bio true daily contact lenses and Acuvue Oasys, Avaira, and Biomedics 55 weekly lenses were able to partially or completely block UV rays under the influence of solar, LED, laser, and UV-A irradiations. Previous studies have shown that the level of UV protection provided by contact lenses is related to certain lens properties, such as diopter power and center thickness [6]. Faubl and Quinn [14] concluded that contact lenses with thin center thickness absorb less UV radiation than thick contact lenses. Recent studies by Ateş and Bilici [15] and Kapfelsberger et al. [16] have confirmed that UV transmittance decreases as the center thickness increases. Furthermore, Artigas et al. [17] evaluated the protective capacity of nine contact lenses against UV radiation emitted from artificial illuminations, such as incandescent, fluorescent, xenon, or white LED lamps. They determined the spectral transmission of the lenses with and without a UV filter and found that some lenses blocked UV radiation due to their different water contents.

The observed differences in the UV protection capacity of the tested lenses may be attributed to variations in lens materials and water content, which were affected by light irradiation [18,19].

Contact lenses should maintain high light transmission to produce high-quality optical images. At the wavelength to which the human eye is most sensitive (550 nm), contact lenses should show maximum light transmission [20].

In this study, the visible light transmittance of contact lenses was reduced when exposed to various light sources. This reduction was observed in most of the tested lenses, including daily and monthly types, as well as Acuvue Oasys and Biomedics 55 weekly types. Laser and UV-A irradiation resulted in a more significant reduction in visible light transmission. These findings have implications for the visual performance of soft contact lenses. Contact lenses must exhibit maximum light transmittance at the visible spectrum, as documented by Rahmani et al. [6]. They studied visible light transmission in some soft contact lenses without the effects of irradiations and reported that all of the studied lenses transmitted at least 94.6% on the visible spectrum.

The effects of different visible and UV-A light sources on daily lens transmissions were conflicting in terms of contact lens transmission in the infrared spectrum. Some lenses and light sources showed increased transmissions, while others showed reduced transmission. Specifically, the Acuvue Oasys and Biomedics 55 Evolution weekly contact lenses exhibited more absorption and reduced transmission under the effect of laser light, while Avaira was more affected by solar light. The irradiation of monthly lenses resulted in increased infrared absorption and reduced transmission. The solar simulator had a significant impact on Biofinity, while PureVision2 was more affected by the LED source. The laser source greatly reduced infrared transmission in the Frequency 55 lens.

Limitations

Only a single example of each lens type was measured, which may limit the generalizability of the findings to a wider range of contact lenses. In addition, the study examines the effects of light exposure in a controlled environment. Real-world factors such as temperature, humidity, and interaction with cleaning solutions may also have effects on contact lenses.

## Conclusions

Solar and artificial lighting, as well as high-powered lasers, significantly affected the transmission and optical properties of contact lenses. All sources of light reduced visible light transmission through the tested contact lenses. Some lenses, such as Bio True Daily Contact Lens, Acuvue Oasys, Avaira, and Biomedics 55 weekly lenses, partially or completely blocked UV transmission. The contact lenses, whether weekly, monthly, or daily, showed varying degrees of infrared transmission. Soft contact lenses with light-protective properties and preserved visible light transmittance are needed.
